# Interim Evaluation of Respiratory Syncytial Virus Hospitalization Rates Among Infants and Young Children After Introduction of Respiratory Syncytial Virus Prevention Products — United States, October 2024–February 2025

**DOI:** 10.15585/mmwr.mm7416a1

**Published:** 2025-05-08

**Authors:** Monica E. Patton, Heidi L. Moline, Michael Whitaker, Ayzsa Tannis, Huong Pham, Ariana P. Toepfer, Christopher A. Taylor, Leah Goldstein, Arthur Reingold, Pam Daily Kirley, Nisha B. Alden, Breanna Kawasaki, James Meek, Daewi Kim, Lucy S. Witt, Kyle P. Openo, Patricia A. Ryan, Erica Mumm, Ruth Lynfield, Yadira Salazar-Sanchez, Francesca Pacheco, Fiona Keating, Bridget J. Anderson, Brenda L. Tesini, Christina B. Felsen, Melissa Sutton, Ann Thomas, William Schaffner, H. Keipp Talbot, Khalil Harbi, Emma Doran, Geoffrey A. Weinberg, Mary A. Staat, Daniel C. Payne, Natasha B. Halasa, Laura Stewart, Julie A. Boom, Leila C. Sahni, Eileen J. Klein, Janet A. Englund, John V. Williams, Marian G. Michaels, Jennifer E. Schuster, Rangaraj Selvarangan, Peter G. Szilagyi, Fiona P. Havers, Fatimah S. Dawood

**Affiliations:** ^1^National Center for Immunization and Respiratory Diseases, CDC; ^2^U.S. Public Health Service Commissioned Corps, Rockville, Maryland; ^3^Eagle Health Analytics, LLC, Atlanta, Georgia; ^4^IHRC, Inc., Atlanta, Georgia; ^5^University of California, Berkeley, Berkeley, California; ^6^California Emerging Infections Program, Oakland, California; ^7^Colorado Department of Public Health & Environment; ^8^Connecticut Emerging Infections Program, Yale School of Public Health, New Haven, Connecticut; ^9^Division of Infectious Diseases, Emory University School of Medicine, Atlanta, Georgia; ^10^Georgia Emerging Infections Program, Georgia Department of Public Health; ^11^Maryland Department of Health, Baltimore, Maryland; ^12^Minnesota Department of Health; ^13^University of New Mexico Health Sciences Center, Albuquerque, New Mexico; ^14^New York State Department of Health; ^15^University of Rochester School of Medicine and Dentistry, Rochester, New York; ^16^Public Health Division, Oregon Health Authority, Portland, Oregon; ^17^Vanderbilt University Medical Center, Nashville, Tennessee; ^18^Division of Public Health, North Carolina Department of Health and Human Services; ^19^Cincinnati Children’s Hospital Medical Center, University of Cincinnati College of Medicine, Cincinnati, Ohio; ^20^Texas Children’s Hospital, Department of Pediatrics, Baylor College of Medicine, Houston, Texas; ^21^Seattle Children’s Research Institute, Seattle, Washington; ^22^UPMC Children’s Hospital of Pittsburgh, University of Pittsburgh School of Medicine, Pittsburgh, Pennsylvania; ^23^University of Missouri, Kansas City School of Medicine, Children’s Mercy Kansas City, Kansas City, Missouri; ^24^UCLA Mattel Children’s Hospital, Los Angeles, California.

**Summary**

**What is already known about this topic?**

Maternal respiratory syncytial virus (RSV) vaccine and nirsevimab, a long-acting monoclonal antibody, help prevent infant RSV-associated hospitalizations; these products became widely available in the United States during the 2024–25 RSV season.

**What is added by this report?**

In this ecologic analysis comparing RSV-associated hospitalization rates among infants aged 0–7 months during 2024–25 with those during pre–COVID-19 pandemic RSV seasons in two surveillance networks, rates during 2024–25 were lower by an estimated 28% and 43%.

**What are the implications for public health practice?**

In the first RSV season with widespread availability of maternal vaccine and nirsevimab, RSV-associated hospitalization rates among infants were lower than in prepandemic seasons. Effective health care planning is needed to protect infants as early in the RSV season as possible through maternal vaccination during pregnancy or infant receipt of nirsevimab.

## Abstract

Maternal respiratory syncytial virus (RSV) vaccine and nirsevimab, a long-acting monoclonal antibody for infants aged 0–7 months and children aged 8–19 months who are at increased risk for severe RSV disease, became widely available for prevention of severe RSV disease among infants and young children during the 2024–25 RSV season. To evaluate the association between availability of these products and infant and child RSV-associated hospitalization rates, the rates among children aged <5 years were compared for the 2024–25 and 2018–20 RSV seasons using data from the RSV-Associated Hospitalization Surveillance Network (RSV-NET) and New Vaccine Surveillance Network (NVSN). Among infants aged 0–7 months (eligible for protection with maternal vaccination or nirsevimab), 2024–25 RSV-associated hospitalization rates were lower compared with 2018–20 pooled rates (estimated relative rate reductions of 43% [RSV-NET: 95% CI = 40%–46%] and 28% [NVSN: 95% CI = 18%–36%]). The largest estimated rate reduction was observed among infants aged 0–2 months (RSV-NET: 52%, 95% CI = 49%–56%; NVSN: 45%, 95% CI = 32%–57%) and during peak hospitalization periods (December–February). These findings support Advisory Committee on Immunization Practices’ recommendations for maternal vaccination or nirsevimab to protect against severe RSV disease in infants and highlight the importance of implementing the recommendations to protect infants as early in the RSV season as possible, before peak transmission, and for infants born during the RSV season, within the first week of life, ideally during the birth hospitalization.

## Introduction

Respiratory syncytial virus (RSV) is the leading cause of hospitalization among U.S. infants, with infants aged 0–2 months at the highest risk for hospitalization (*1*). Two effective products for preventing infant RSV hospitalizations, maternal RSV vaccine^§^ (*2*) (administered during weeks 32–36 of pregnancy during September–January in most of the United States) and nirsevimab^¶^ (*3*) (a long-acting monoclonal antibody for all infants aged 0–7 months in their first RSV season and children aged 8–19 months in their second RSV season at increased risk for severe RSV disease) were introduced during the 2023–24 U.S. RSV season**^,^^††^ (*2*–*5*). To assess a possible association between availability of these products and RSV-associated hospitalizations, this ecologic analysis compared pediatric RSV-associated hospitalization rates from two U.S. active surveillance systems, the RSV-Associated Hospitalization Surveillance Network (RSV-NET) and New Vaccine Surveillance Network (NVSN), during 2024–25 and 2018–20.

## Methods

### Data Sources

RSV-NET conducts active population-based surveillance for laboratory-confirmed^§§^ RSV-associated hospitalizations identified through clinical testing among catchment-area residents of all ages in approximately 300 hospitals in 161 counties across 13 states.^¶¶^^,^*** NVSN conducts active, population-based surveillance for acute respiratory illness (ARI) among hospitalized children aged <18 years at seven U.S. medical centers^†††^ (*4*); respiratory specimens from all enrolled children are tested for RSV.^§§§^ RSV-NET and NVSN have both used standardized case definitions and active case finding since 2016 and 2000, respectively (*4*,*6*), and both collect demographic data through medical record abstraction. NVSN also conducts parent interviews.

This analysis included RSV-NET and NVSN RSV-associated hospitalization data from children aged <5 years during the 2018–19 and 2019–20 RSV seasons (October–April, representing typical RSV seasons before the COVID-19 pandemic) and the 2024–25 season (October–February data); 2018–19 and 2019–20 data were pooled to generate 2018–20 data. The 2020–21 through 2023–24 RSV seasons were excluded because the COVID-19 pandemic resulted in atypical RSV seasonality and circulation (*7*).

### Data Analysis

Hospitalization rates for three groups with varying eligibility for RSV prevention products were analyzed: 1) infants aged 0–7 months eligible for protection through maternal RSV vaccine or nirsevimab, overall and in prespecified subgroups of infants aged 0–2 months, who are at highest risk for RSV-associated hospitalization (*1*) and infants aged 3–7 months; 2) children aged 8–19 months entering their second RSV season, some of whom might have been nirsevimab-eligible based on risk conditions^¶¶¶^; and 3) children aged 20–59 months, who were ineligible for either product. Weekly (RSV-NET)**** or monthly (NVSN)^††††^ RSV-associated hospitalizations per 1,000 children aged <5 years were calculated using U.S. population denominators (*4*,*6*); cumulative rates for all seasons were estimated through February to ensure consistent comparisons. RSV-NET rates were adjusted to account for RSV underdetection related to testing practices and test sensitivity using an established multiplier approach^§§§§^ (*6*,*8*). NVSN rates were adjusted for enrollment rates, weeks with <7 surveillance days, test sensitivity, and hospital market share^¶¶¶¶^ (*4*). A sensitivity analysis excluding the Houston, Texas site from NVSN data (approximately 20% of overall enrolled children) was performed because RSV circulation and associated hospitalizations increased in Houston in September 2024, before RSV prevention products were widely administered there (Supplementary Figure).

Rate ratios (RRs) and 95% CIs were estimated, and differences were assessed with Z-tests or *t*-tests comparing cumulative 2024–25 hospitalization rates with pooled 2018–20 rates.***** Relative hospitalization rate reduction (RRR) was estimated as (1 − RR) x 100. Trends and differences by age were compared using Cochran-Armitage trends and Pearson’s chi-square tests, respectively. Data were analyzed using SAS (version 9.4; SAS Institute). This activity was reviewed by CDC, deemed not research, and conducted consistent with applicable federal law and CDC policy.^†††††^

## Results

### Characteristics of Children Hospitalized for RSV

Overall, 18,389 RSV-associated hospitalizations (15,405 in RSV-NET and 2,984 in NVSN) were identified among children aged <5 years; these included 11,681 during 2018–20 and 6,708 during 2024–25. Median patient age was 6.7 months and 14.7 months in RSV-NET (p<0.001) and 6.3 months and 12.7 months in NVSN (p<0.04), in the earlier versus later seasons, respectively (Supplementary Table 1).

### RSV-Associated Hospitalization Rates Among Infants Aged 0–7 Months

Cumulative RSV-associated hospitalization rates among infants aged 0–7 months were lower during 2024–25 compared with 2018–20: 8.5 versus 15.0 per 1,000 children in RSV-NET, and 10.7 versus 14.8 per 1,000 children in NVSN (Table). These differences were associated with estimated hospitalization rate reductions of 43% in RSV-NET and 28% in NVSN during 2024–25 (p<0.001 for both). Estimated rate reductions were largest during December–February (Supplementary Table 2). In a sensitivity analysis of NVSN data excluding Houston (because of earlier RSV season onset before prevention product availability), the apparent reduction in RSV-associated hospitalization rates among infants aged 0–7 months during 2024–25 was larger (56%) (Table).

**TABLE:** Hospitalization rates among U.S. children aged <5 years with laboratory-confirmed respiratory syncytial virus — Respiratory Syncytial Virus–Associated Hospitalization Surveillance Network and New Vaccine Surveillance Network, United States, 2018–20 and 2024–25

Surveillance system/RSV season/Hospitalization metrics	Age group, mos				
	0–7			8–19	20–59
	All (0–7)	0–2	3–7		
RSV-NET*					
**2018–20**					
Hospitalizations^†^ (n = 9,547), no. (%)	4,857 (51)	2,694 (28)	2,163 (23)	2,428 (25)	2,262 (24)
Pooled hospitalization rate^§^ (95% CI)	15.0 (14.7 to 15.4)	22.2 (21.5 to 22.9)	10.7 (10.3 to 11.1)	5.0 (4.9 to 5.2)	1.5 (1.5 to 1.6)
**2024–25**					
Hospitalizations^†^ (n = 5,858), no. (%)	1,623 (28)	755 (13)	868 (15)	1,935 (33)	2,300 (39)
Hospitalization rate^§^ (95% CI)	8.5 (8.1 to 8.9)	10.6 (9.9 to 11.3)	7.3 (6.8 to 7.8)	6.7 (6.4 to 7.0)	2.5 (2.4 to 2.6)
Rate ratio (95% CI)^¶^	0.57 (0.54 to 0.60)	0.48 (0.44 to 0.51)	0.68 (0.63 to 0.73)	1.33 (1.26 to 1.41)	1.64 (1.56 to 1.72)
P-value^¶^	<0.001	<0.001	<0.001	<0.001	<0.001
RRR, % (95% CI)**^,††^	43 (40 to 46)	52 (49 to 56)	32 (27 to 37)	–33 (–41 to –26)	–64 (–72 to –56)
**NVSN^§§^**					
**2018–20**					
Hospitalizations^†^ (n = 2,134), no. (%)	1,204 (56)	659 (31)	545 (25)	517 (24)	413 (20)
Pooled hospitalization rate^§^ (95% CI)	14.8 (14.0 to 15.6)	21.7 (20.0 to 23.3)	10.6 (9.7 to 11.5)	4.7 (4.3 to 5.1)	1.1 (1.0 to 1.2)
**2024–25**					
Hospitalizations^†^ (n = 850), no. (%)	311 (37)	128 (15)	183 (22)	295 (35)	244 (28)
Hospitalization rate^§^ (95% CI)	10.7 (9.4 to 12.0)	12 (9.8 to 14.4)	9.9 (8.5 to 11.3)	5.9 (5.2 to 6.7)	1.7 (1.5 to 1.9)
Rate ratio (95% CI)^¶^	0.72 (0.64 to 0.82)	0.55 (0.43 to 0.68)	0.93 (0.77 to 1.10)	1.26 (1.08 to 1.46)	1.63 (1.36 to 1.90)
P-value^¶^	<0.001	<0.001	0.56	0.02	<0.001
RRR, % (95% CI)**^,††^	28 (18 to 36)	45 (32 to 57)	7 (–10 to 23)	–26 (–46 to –8)	–63 (–90 to –36)
**NVSN excluding Houston, Texas**					
**2018–20**					
Hospitalizations^†^ (n = 1,721), no. (%)	978 (57)	530 (31)	448 (26)	405 (23)	338 (20)
Pooled hospitalization rate^§^ (95% CI)	19 (17.8 to 20.1)	26.4 (24.4 to 28.6)	14.6 (13.3 to 15.8)	6.0 (5.4 to 6.6)	1.6 (1.5 to 1.8)
**2024–25**					
Hospitalizations^†^ (n = 698), no. (%)	223 (32)	87 (13)	136 (19)	237 (34)	238 (34)
Hospitalization rate^§^ (95% CI)	8.4 (7.3 to 9.6)	7.6 (5.8 to 9.6)	8.9 (7.5 to 10.3)	6.4 (5.6 to 7.2)	2.3 (2.0 to 2.6)
Rate ratio (95% CI)^¶^	0.44 (0.38 to 0.51)	0.29 (0.22 to 0.36)	0.62 (0.50 to 0.73)	1.07 (0.90 to 1.24)	1.42 (1.19 to 1.67)
P-value^¶^	<0.001	<0.001	<0.001	0.51	<0.001
RRR, % (95% CI)**^,††^	56 (49 to 62)	71 (64 to 78)	38 (27 to 50)	–7 (–24 to 10)	–42 (–67 to –19)

**Abbreviations**: NVSN = New Vaccine Surveillance Network; RRR = relative hospitalization rate reduction; RSV = respiratory syncytial virus; RSV-NET = Respiratory Syncytial Virus–Associated Hospitalization Surveillance Network.

* RSV-NET conducts active population-based surveillance for laboratory-confirmed RSV-associated hospitalizations identified through clinical testing among catchment-area residents of all ages in approximately 300 hospitals in 161 selected counties in California, Colorado, Connecticut, Georgia, Maryland, Michigan, Minnesota, New Mexico, New York, North Carolina, Oregon, Tennessee, and Utah.

^†^ Hospitalization data were included from October–April 2018–20 and October–February 2024–25.

^§^ Cumulative laboratory-confirmed RSV-associated hospitalizations per 1,000 children aged <5 years as of February 28 each season. Rates use U.S. population denominators and are adjusted to account for RSV underdetection because of testing practices and test sensitivity.

^¶^ Z-tests (RSV-NET) or *t*-tests (NVSN) were used to assess whether rate ratios differed from 1, with p<0.05 considered statistically significant.

** RRR was estimated as (1 – rate ratio) x 100%.

^††^ RRR 95% CIs that excluded 0 were considered statistically significant, corresponding to rate ratio 95% CIs excluding 1 and p<0.05.

^§§^ NVSN conducts active, population-based surveillance for acute respiratory illness among hospitalized children aged <18 years at seven U.S. medical centers. NVSN population-based RSV-associated hospitalization rates were generated using actively enrolled residents of defined catchment-area counties for the seven sites that were included: Cincinnati (Hamilton County, Ohio); Houston (Brazoria, Chambers, Fort Bend, Galveston, Harris, Liberty, Montgomery, and Waller counties, Texas); Kansas City (Jackson County, Missouri); Nashville (Davidson County, Tennessee); Pittsburgh (Allegheny County, Pennsylvania); Rochester (Monroe County, New York); and Seattle (King County, Washington).

The largest cumulative rate differences during 2024–25 compared with 2018–20 were observed among infants aged 0–2 months. Estimated reductions in RSV-associated hospitalization rates among infants aged 0–2 months were 52% for RSV-NET and 45% for NVSN (p<0.001 for all); the NVSN rate reduction was larger (71%) with Houston excluded.

### RSV-Associated Hospitalization Rates Among Children Aged 8–19 and 20–59 Months

**FIGURE 1:**
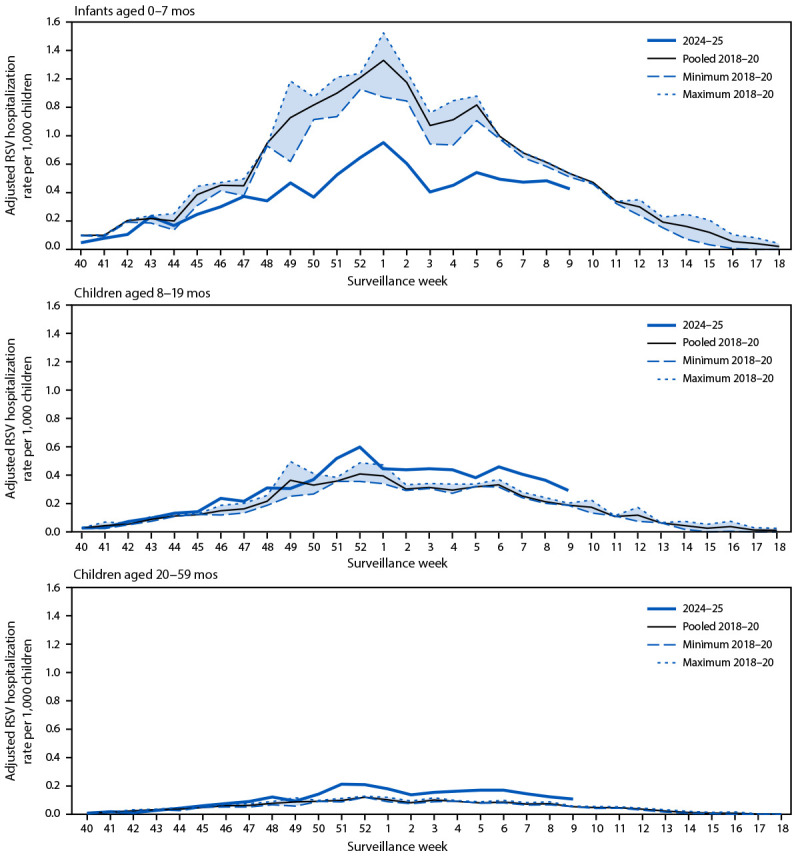
Respiratory syncytial virus–associated hospitalization rates* among children aged <5 years, by age group and surveillance week — Respiratory Syncytial Virus–Associated Hospitalization Surveillance Network, United States, October–April 2018–20 and October–February 2024–25 **Abbreviation:** RSV = respiratory syncytial virus. * Rate of laboratory-confirmed RSV–associated hospitalizations identified through clinical testing among catchment-area residents per 1,000 children aged <5 years. Rates use U.S. population denominators and are adjusted to account for RSV underdetection because of testing practices and test sensitivity. Pooled rates from 2018 through 2020 were estimated by dividing total RSV hospitalizations in the 2018–19 and 2019–20 RSV seasons by pooled population estimates; minimum and maximum weekly rates reflect the lowest and highest observed rates, respectively, for each week across the 2018–19 and 2019–20 seasons. Data for the 2024–25 RSV season were only available through February 2025.

**FIGURE 2:**
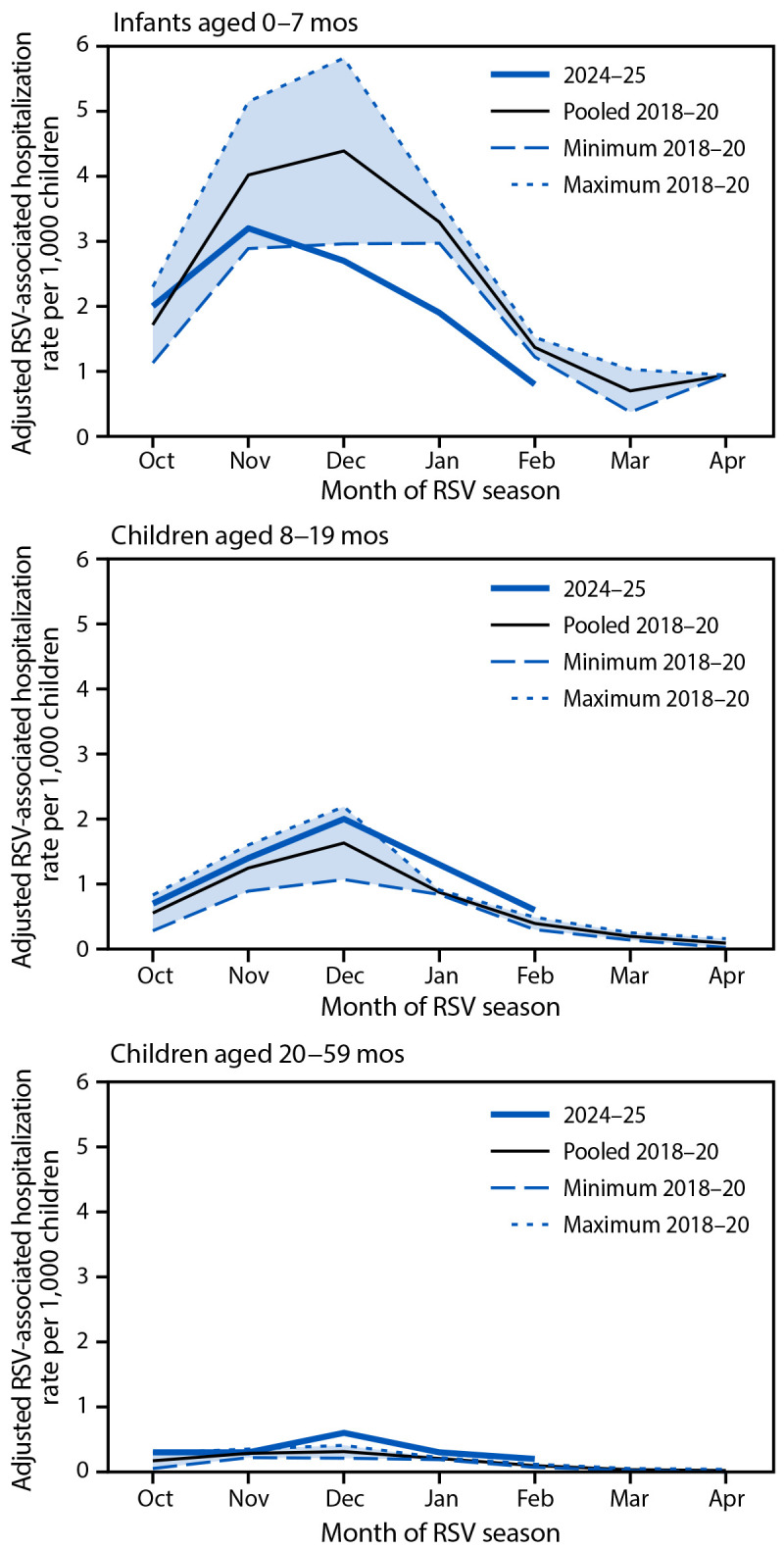
Respiratory syncytial virus–associated hospitalization rates* among children aged <5 years, by age group and month of respiratory syncytial virus season — New Vaccine Surveillance Network, United States, October–April 2018–20 and October–February 2024–25 **Abbreviation:** RSV = respiratory syncytial virus. * Rate of laboratory-confirmed RSV-associated hospitalizations among all catchment-area residents, including Houston, per 1,000 children aged <5 years. Rates use U.S. population denominators and are adjusted for enrollment rates, weeks with <7 surveillance days, test sensitivity, and hospital market share. Pooled rates from 2018 through 2020 were estimated by dividing total RSV hospitalizations in the 2018–19 and 2019–20 RSV seasons by pooled population estimates; minimum and maximum monthly rates reflect the lowest and highest observed rates, respectively, for each month across the 2018–19 and 2019–20 seasons. Data for the 2024–25 RSV season were only available through February 2025.

Among children aged 8–19 and 20–59 months, RSV-associated hospitalization rates were higher during 2024–25 than during 2018–20. Differences in weekly and monthly rates between 2024–25 and 2018–20 among all age groups were comparable to observed differences in cumulative rates (Figure 1) (Figure 2).

## Discussion

In 2024–25, the first U.S. RSV season with widespread availability of maternal RSV vaccine and nirsevimab, analyses of two population-based surveillance networks demonstrated significantly lower RSV-associated hospitalization rates among infants aged 0–7 months who were eligible for RSV prevention products, with estimated rate reductions of 28% and 43% compared with rates during the pooled 2018–20 RSV seasons. The largest estimated rate reductions in hospitalization occurred among infants aged 0–2 months (*1*).

Higher RSV-associated hospitalization rates during 2024–25 compared with 2018–20 among children in older age groups, who were largely ineligible for RSV prevention products, suggest a more severe 2024–25 season overall and indicate that observed reductions in hospitalization rates among younger infants might be underestimated. Increased hospitalization rates among these older children also suggest that reduced infant hospitalization rates were likely due to RSV prevention products, rather than to changes in RSV circulation, testing practices, or health care–seeking behavior. The apparent reduction in RSV-associated infant hospitalization rates temporally associated with widespread availability of two options to protect eligible infants (i.e., maternal RSV vaccination and nirsevimab administration to eligible infants) suggests that most severe RSV disease among infants aged 0–7 months is preventable, consistent with findings in European countries (*9*,*10*). In this analysis, rate decreases were largest among infants aged 0–2 months, the group at highest risk for RSV-associated hospitalization (*1*). The findings suggest the importance of protecting infants born during the RSV season through either maternal vaccination during pregnancy or nirsevimab administration in the first week of life, ideally during the birth hospitalization (*2*).

National immunization survey data indicate the estimated proportion of U.S. infants aged 0–7 months protected by either maternal vaccination or nirsevimab increased during the 2024–25 RSV season, from 30% in October 2024 to 66% in February 2025,^§§§§§^ coinciding with the 2024–25 RSV-associated hospitalization rate reductions in both surveillance networks, with the largest monthly reductions occurring during peak hospitalization periods. In addition, reduction in hospitalization rates among NVSN infants aged 0–7 months were larger after excluding Houston, where prevention products were not widely available before RSV season onset. These results support the recommendations of the Advisory Committee on Immunization Practices to optimize population-level impact by administering RSV prevention products as early as possible in the season (i.e., before peak RSV transmission) on the basis of local epidemiology (*3*). Increased and earlier use of RSV prevention products during future seasons might lead to even larger reductions in pediatric RSV–associated hospitalizations.

### Limitations

The findings in this report are subject to at least four limitations. First, this was an ecologic analysis and does not include individual-level data on coverage with RSV prevention products; therefore, causality could not be assessed. Second, hospitalization rate adjustments accounting for RSV underdetection or underenrollment might be insufficient. Third, RSV-NET and NVSN catchment areas might not be nationally representative. Finally, interim results might underestimate changes during complete RSV seasons or seasons with higher product coverage. However, relatively consistent findings from two geographically diverse, population-based surveillance networks provide reliable support for the population-level impacts of RSV prevention products on U.S. pediatric RSV-associated hospitalizations.

### Implications for Public Health Practice

During the first RSV season with widespread availability of prevention products, RSV-associated hospitalization rates were significantly lower compared with those during pre–COVID-19 pandemic seasons among infants aged 0–7 months. Reductions were largest during peak hospitalization periods. These findings highlight the importance of effective annual health care planning to implement Advisory Committee on Immunization Practices’ recommendations for RSV prevention products and ensure parents can protect infants as early as possible in the RSV season, either through maternal vaccination during pregnancy or infant receipt of nirsevimab. For infants born during the RSV season who are not protected through maternal vaccination, nirsevimab should be administered within the first week of life, ideally during the birth hospitalization.

## Footnotes

* These authors contributed equally to this report.

§ Maternal RSV vaccine should be administered to pregnant women at 32–36 weeks’ gestation during September–January in most of the continental United States to protect infants during their first months of life when maternal vaccination protection is highest and during the RSV season when RSV circulation is highest. Administering vaccine starting in September (1–2 months before anticipated RSV season start) through January (2–3 months before anticipated RSV season end) maximizes cost-effectiveness and benefits. In jurisdictions with RSV seasonality that differs from the continental United States, providers should follow state, local, or territorial guidance on timing.

¶ Beginning shortly before the RSV season starts, providers should administer nirsevimab to infants aged <8 months and to children aged 8–19 months at increased risk for severe RSV disease. Nirsevimab could be administered in most of the continental United States from October through the end of March. Because timing of onset, peak, and decline of RSV activity might vary geographically, providers can adjust administration schedules based on local epidemiology. Providers should consult state, local, or territorial guidance on timing of nirsevimab administration.

**
Nirsevimab Coverage, Children 0 to 7 months, United States | RSVVaxView | CDC

††
Infant Protection Against Respiratory Syncytial Virus (RSV) by Maternal RSV Vaccination or Receipt of Nirsevimab, and Intent for Nirsevimab Receipt, United States

§§ RSV-associated hospitalizations are defined as those among persons who have received a positive RSV reverse transcription–polymerase chain reaction (RT-PCR) or rapid antigen detection test result ≤14 days before or after hospital admission.

¶¶ RSV-NET population-based RSV-associated hospitalization rates were generated for the 2018–19 and 2019–20 seasons for residents in 65 and 72 selected counties and county equivalents, respectively, in California, Colorado, Connecticut, Georgia, Maryland, Michigan, Minnesota, New Mexico, New York, Oregon, Tennessee, and Utah; for the 2024–25 season, rates were generated for residents from 161 selected counties and county equivalents in these states and North Carolina.

***
RSV-NET | RSV | CDC

††† NVSN population-based RSV-associated hospitalization rates were generated using actively enrolled residents of defined catchment area counties for the seven sites that were included: Cincinnati (Hamilton County, Ohio); Houston (Brazoria, Chambers, Fort Bend, Galveston, Harris, Liberty, Montgomery, and Waller counties, Texas); Kansas City (Jackson County, Missouri); Nashville (Davidson County, Tennessee); Pittsburgh (Allegheny County, Pennsylvania); Rochester (Monroe County, New York); and Seattle (King County, Washington).

§§§ Systematically collected respiratory specimens were tested by real-time RT-PCR.

¶¶¶ Infants and children aged 8–19 months at increased risk for severe RSV disease include those with chronic lung disease of prematurity who require medical support (chronic corticosteroid therapy, diuretic therapy, or supplemental oxygen), severe immunocompromise, or cystic fibrosis who have manifestations of severe lung disease or weight-for-length <10th percentile; and children of American Indian or Alaska Native descent.

**** Unadjusted RSV-NET hospitalization rates (hospitalizations per 1,000 children aged <5 years) were estimated by dividing total catchment-area RSV hospitalizations by National Center for Health Statistics (NCHS) population estimates; population estimates were divided by 12 to estimate rates by month of life. NCHS vintage 2020 bridge-race postcensal population estimates were used for seasons 2018–19 to 2019–20; U.S. Census Bureau vintage unbridged-race postcensal estimates were used for October 2020 and later.

†††† For the NVSN 2018–20 rate analysis, county-specific population denominator data were obtained from the 2020 U.S. bridged-race population estimates. The 2024–25 rate analysis used 2022 interim population estimates because these were the most recent available data. Children with inconclusive RSV testing results were excluded. Children with RSV detection from a rapid test but no detection from a molecular test were excluded because of poor rapid test specificity. Population-based numerators were calculated by adjusting the observed number of hospitalizations to account for weeks with <7 days of surveillance, the percentage of eligible children not enrolled, sensitivity of RSV RT-PCR testing (87.6%, using serology as the standard relative to RT-PCR), and the market share of each enrollment hospital site. Rates were estimated per 1,000 children aged <5 years, and 95% CIs were determined by bootstrap percentiles based on 1,000 bootstrap samples for each rate.

§§§§ RSV-NET only captures children hospitalized with RSV if they were tested for RSV and if tests accurately detect RSV. To correct for RSV hospitalization underascertainment, each RSV-NET site identified all children aged <5 years hospitalized during 2018–2023 with ARI in select hospitals, as identified by *International Classification of Diseases, Tenth Revision, Clinical Modification* codes, and determined the proportion of those children who received testing for RSV and type of RSV test used among a stratified random sample. Adjustment multipliers, the inverse of RSV testing frequency times the average test sensitivity (using 74% sensitivity for rapid antigen tests and 96% for molecular diagnosis), were estimated for each season using testing data from hospitalizations in each age group. Adjusted hospitalization rates were calculated by multiplying unadjusted rates by adjustment multipliers and presented with 95% CIs to account for multiplier uncertainty; 2022–23 adjustment multipliers were the most recent available data and applied to 2024–25. Rate ratios, 95% CIs, and Z-tests were used to identify significant differences between rates.

¶¶¶¶ NVSN population-based numerators are calculated by adjusting the observed number of hospitalizations to account for weeks with <7 days of surveillance, the percentage of eligible children not enrolled, sensitivity of RSV RT-PCR testing (87.6%), and the market share of each enrollment hospital site for the estimated proportion of catchment-area ARI hospitalizations captured.

***** Pooled rates from 2018 through 2020 were estimated by dividing total RSV hospitalizations in the 2018–19 and 2019–20 seasons by pooled population estimates.

††††† 45 C.F.R. part 46.102(l)(2), 21 C.F.R. part 56; 42 U.S.C. Sect. 241(d); 5 U.S.C. Sect. 552a; 44 U.S.C. Sect. 3501 et seq.

§§§§§
Infant Protection Against Respiratory Syncytial Virus (RSV) by Maternal RSV Vaccination or Receipt of Nirsevimab, and Intent for Nirsevimab Receipt, United States
